# Nutritional status and bacteremia patterns in children with diarrheal diseases: A comparative analysis of bacteremia from *Salmonella* Typhi versus other pathogens

**DOI:** 10.1371/journal.pone.0333580

**Published:** 2025-10-08

**Authors:** Md Rezaul Hossain, Monira Sarmin, Irin Parvin, Mst Mahmuda Ackhter, Afsan Bulbul, Chidozie Declan Iwu, Mohammod Jobayer Chisti, Lubaba Shahrin

**Affiliations:** 1 Department of Epidemiology and Biostatistics, School of Public Health, Texas A&M University, College Station, Texas, United States of America; 2 Nutrition Research Division, icddr, b, Dhaka, Bangladesh; 3 Royal Blackburn Teaching Hospital, Blackburn, United Kingdom; 4 Department of Epidemiology, School of Public Health, University of Washington, Seattle, Washington, United States of America; Dana-Farber Cancer Institute, UNITED STATES OF AMERICA

## Abstract

**Background:**

Bacteremia remains a significant concern among under-five children with diarrheal diseases, particularly in resource-limited settings. Distribution of bacteremia patterns across the patient’s nutritional status and outcomes have never been analyzed. This study aimed to investigate the association between nutritional status and bloodstream infections caused by *Salmonella enterica* serovar Typhi compared to other pathogenic bacteria in children with diarrheal diseases.

**Methods:**

A retrospective case-control study was conducted using electronic medical records from icddr,b (Dhaka, Bangladesh) between 2019−20. Cases were defined as children (< 60 months) hospitalized with diarrheal disease and diagnosed with *Salmonella* Typhi bacteremia; controls included children with bloodstream infections caused by other than typhoidal bacteria, including *Escherichia coli, Klebsiella pneumoniae, Pseudomonas aeruginosa*, and *Streptococcus* spp. Nutritional status was categorized as well-nourished, Moderate Acute Malnutrition (MAM), or Severe Acute Malnutrition (SAM). Descriptive statistics and multiple logistic regression models were used to assess associations between nutritional status, bacteremia type, and clinical outcomes.

**Results:**

Among 162 children with confirmed bloodstream infections, 74 (45.68%) had *Salmonella* Typhi bacteremia, while 88 (54.32%) had bacteremia caused by other bacterial isolates. SAM was more prevalent among children with other bacteremia (78.12%) than caused by *Salmonella* Typhi. Conversely, well- nourished children were more likely to develop *Salmonella* Typhi bacteremia (66.13%) compared to MAM (32.61%) and SAM (21.88%) cases. After adjusting for comorbidities and prior antibiotics use, logistic regression analysis found malnourished children had significantly lower odds of developing *Salmonella* Typhi bacteremia compared to well-nourished children (SAM: aOR 0.157, 95% CI: 0.045–0.548, p = 0.004; MAM: aOR 0.238, 95% CI: 0.089–0.640, p = 0.004). Mortality rates were significantly higher among controls (11.73%) compared to *Salmonella* Typhi cases (1.35%), particularly for infections caused by *Klebsiella pneumoniae* (66.67%) and *E. coli* (31.25%).

**Conclusion:**

Malnourished children are at higher risk for severe bloodstream infections caused by other bacterial species, leading to higher mortality rates and increased antimicrobial resistance. However, *Salmonella* Typhi bacteremia occurred more frequently in well-nourished children. These sort of distribution of bacteremia patterns across patients’ nutritional status can provide insights and improve clinical management.

## Introduction

Diarrheal diseases remain a significant cause of morbidity and mortality in under-five children, particularly in low- and middle-income countries [1]. Among the many pathogens contributing to diarrhea-associated complications, *Salmonella* Typhi is a major concern due to its ability to cause systemic infections, particularly typhoid fever [2]. Globally, typhoid fever remains a significant health concern, with the highest incidence rates among under-five children observed in the WHO South-East Asian, Eastern Mediterranean, and African regions, where the incidence can reach up to 306 cases per 100,000 persons [2]. Bacteremia, where bacteria invade the bloodstream, is a severe complication of typhoid fever that can lead to life-threatening outcomes if not promptly treated [3,4].

Beyond *Salmonella* Typhi, several other pathogenic bacteria, including Non-Typhoidal *Salmonella*, *Escherichia coli*, *Streptococcus* spp.*, Staphylococcus* spp., Methicillin-Resistant *Staphylococcus aureus* (MRSA), *Klebsiella* spp., *Pseudomonas* spp*.*, and *Acinetobacter* spp*.*, are also frequently associated with bloodstream infections in pediatric populations [2]. These pathogens often contribute to severe infections, more frequently in malnourished children who are already immunocompromised [2,5–7]. While the impact of nutritional deficiencies on susceptibility to infections is well-documented, the specific interplay between malnutrition and bloodstream infections due to different bacterial pathogens remains unclear [8–10].

Previous studies indicate that children with severe acute malnutrition (SAM) and moderate acute malnutrition (MAM) are at heightened risk for invasive bacterial infections due to immune suppression [11,12]. While some studies suggest that malnourished children may have an increased risk of developing *Salmonella* Typhi bacteremia [13,14], other studies found that malnutrition predisposes children more toward infections caused also by other invasive bacterial pathogens including Non-Typhoidal *Salmonella, Escherichia coli, Streptococcus* spp., *Staphylococcus* spp., MRSA, *Klebsiella* spp., *Pseudomonas* spp., and *Acinetobacter* spp. [2,15–17]. There are no studies that have documented any differences in nutritional status or other clinical factors between typhoidal bacteremia and other types of bacteremia.

This study compares the prevalence and characteristics of *Salmonella* Typhi bacteremia to other bacterial bloodstream infections in children with diarrheal diseases. We analyze nutritional status, comorbidities, and bacterial isolates to determine if malnourished children are more vulnerable to severe infections from other bacteremia while well-nourished children are disproportionately affected by *Salmonella* Typhi bacteremia.

## Materials and methods

A retrospective case-control study was conducted (from January 2018 to June 2019) using International Centre for Diarrhoeal Disease Research, Bangladesh (icddr,b) – Dhaka hospital’s electronic medical records to compare children diagnosed with *Salmonella* Typhi bacteremia to those with other bacterial bloodstream infections than typhoidal (*Salmonella* Typhi).

Cases were defined as children admitted with diarrheal diseases (acute diarrhea (≥3 loose stools per day), persistent diarrhea (>14 days), or dysentery, as per WHO criteria) [2] who were diagnosed with *Salmonella* Typhi bacteremia confirmed from blood culture during 2019−20. Controls were children admitted with diarrheal diseases who were found to have bloodstream infections caused by other pathogenic bacterial species, including Non-Typhoidal *Salmonella, Escherichia coli, Streptococcus* spp., *Staphylococcus* spp., MRSA, *Klebsiella* spp., *Pseudomonas* spp., and *Acinetobacter* spp.

Inclusion criteria: (1) Children under five years of age; (2) Admitted with diarrheal diseases; (3) Underwent blood culture testing for suspected bacteremia and then confirmed; (4) Complete records of anthropometric, laboratory, and clinical parameters.

Exclusion criteria: (1) Children with incomplete medical records; (2) Cases of viral or fungal bloodstream infections; (3) Patients with known primary immunodeficiency disorders. Children were excluded if they had missing (confirmed) blood culture results, missing anthropometric data, or incomplete clinical records. Additionally, participants with no bacterial growth or skin contaminants were also excluded.

The primary exposure variable was nutritional status, categorized as well-nourished, MAM, and SAM based on WHO-defined anthropometric criteria [5]. Other covariates included age, sex, presence of comorbidities, prior antibiotic use, and clinical outcomes.

Blood samples were collected and processed at the icddr,b microbiology laboratory. Blood cultures were performed using automated systems to identify bacterial growth. Antibiotic susceptibility testing was conducted according to Clinical and Laboratory Standards Institute (CLSI) guidelines.

Descriptive statistics were used to compare baseline characteristics between cases and controls. Categorical variables were analyzed using chi-square tests. Logistic regression models were applied to estimate adjusted odds ratios (aORs) and 95% confidence intervals (CIs) for the association between nutritional status and *Salmonella* Typhi bacteremia, adjusting for potential confounders such as age, sex, comorbidities (pneumonia, sepsis, and other systemic infections), and prior antibiotic use. A p-value of <0.05 was considered statistically significant.

Ethical approval was obtained from the institutional review board (IRB) of icddr,b. As this study used de-identified patient data, individual consent was waived from the icddr,b IRB.

## Results

A total of 162 children with confirmed bloodstream infections were included in the study for analysis, with 74 cases of *Salmonella* Typhi bacteremia and 88 cases of bacteremia caused by including Non-Typhoidal *Salmonella, Escherichia coli, Streptococcus* spp., *Staphylococcus* spp., MRSA, *Klebsiella* spp., *Pseudomonas* spp., and *Acinetobacter* spp., serving as controls. The distribution of age, sex, nutritional status, comorbidities (pneumonia, sepsis, and other systemic infections), and prior antibiotic resistance use was analyzed to assess their association with typhoid versus other bacterial bloodstream infections.

The median age of children with *Salmonella* Typhi bacteremia was significantly higher (23.12 months, IQR: 15.3–36.3) compared to the control group (8.62 months, IQR: 4.88–12.84). Nutritional status differed between the two groups. Among cases, 66.13% were well-nourished, while only 33.87% of the controls were well-nourished (Table 1). In contrast, SAM was more prevalent among controls (78.12%) than in cases (21.88%), indicating that children with severe malnutrition were more prone to bacteremia caused by bacterial infections other than *Salmonella* Typhi.

**Table 1:** Baseline characteristics of study participants.

Characteristic	Total, N (%)	*Salmonella* Typhi Bacteremia (Cases) (n = 74)	Other Bacteremia (Controls) (n = 88)	p-value
Age (months, median [range])	16.65 (4.88-36.3)	23.12 (15.3-36.3)	8.62 (4.88-12.84)	<0.001
Male (%)	89 (54.94)	54.05%	55.68%	0.842
Well-nourished (%)	62 (38.27)	66.13%	33.87%	<0.001
Moderate Acute Malnutrition (MAM) (%)	46 (28.4)	32.61%	67.39%	<0.001
Severe Acute Malnutrition (SAM) (%)	32 (19.75)	21.88%	78.12%	<0.001
Comorbidities^*^ (%)	73 (45.06)	25.67%	61.36%	<0.001
Antibiotics used prior admission	118 (72.84)	79.55%	67.57%	0.074

* Pneumonia, sepsis, and other systemic infections.

While among total, about near half (45%) had comorbidities, a significantly higher prevalence of comorbidities was observed among controls (61.36%) compared to cases (25.67%). The most common comorbidities included pneumonia, sepsis, and other systemic infections. The severity of illness among the control group was reflected in longer hospital stays and increased need for intensive care support. Antibiotic use before obtaining blood culture was higher among controls (79.55%) compared to cases (67.57%).

Among all bacteremia cases, *Salmonella* Typhi was the most frequently isolated pathogen (45.68%), followed by *Escherichia coli* (9.88%), *Streptococcus* spp. (6.79%), *Pseudomonas* spp. (6.79%), and *Klebsiella* spp. (3.70%) (Table 2). This distribution suggests that malnourished children were at greater risk of infections caused by opportunistic and highly resistant pathogens rather than *Salmonella* Typhi alone.

**Table 2:** Distribution of bacterial pathogens by mortality outcomes.

Organism	Total (%)	Death (%)	Survival (%)	OR (95% CI)	p-value (<0.005*)
*Salmonella* Typhi	74 (45.7)	1 (1.4%)	73 (98.6%)	0.05 (0.01-0.41)	0.004*
Non-Typhoidal *Salmonella*	8 (4.9)	1 (12.5%)	7 (87.5%)	1.08 (0.13-9.29)	0.945
*E. coli*	16 (9.9)	5 (31.3%)	11 (68.8%)	4.29 (1.30-14.12)	0.017
*Streptococcus* sp.	11 (6.8)	0 (0%)	11 (100%)	–	–
*Staphylococcus* sp.	6 (3.7)	0 (0%)	6 (100%)	–	–
MRSA	8 (4.9)	2 (25%)	6 (75%)	3.04 (0.56-16.45)	0.196
*Klebsiella* sp.	6 (3.7)	4 (66.7%)	2 (33.3%)	18.80 (3.17-111.36)	0.001*
*Pseudomonas* sp.	11 (6.8)	4 (36.4%)	7 (63.6%)	5.18 (1.36-19.77)	0.016
*Acinetobacter* sp.	11 (6.8)	0 (0%)	11 (100%)	–	–
Less Found Organisms**	11 (6.8)	2 (18.2%)	9 (81.8%)	1.75 (0.35-8.79)	0.496

Less Found Organisms** include *Campylobacter* sp., *Enterobacter* sp., *Aeromonas hydrophila, Burkholderia cepacian*, *Hemophilus influenza*, and *Raoultella ornithinolytica*.

Mortality rates were significantly higher among controls (11.73%) compared to cases (1.35%). The highest case fatality rates were observed in infections caused by *Klebsiella pneumoniae* (66.67%). The increased mortality in the control group underscores the severity of infections caused by opportunistic bacterial pathogens, especially in malnourished children.

**Fig 1:**
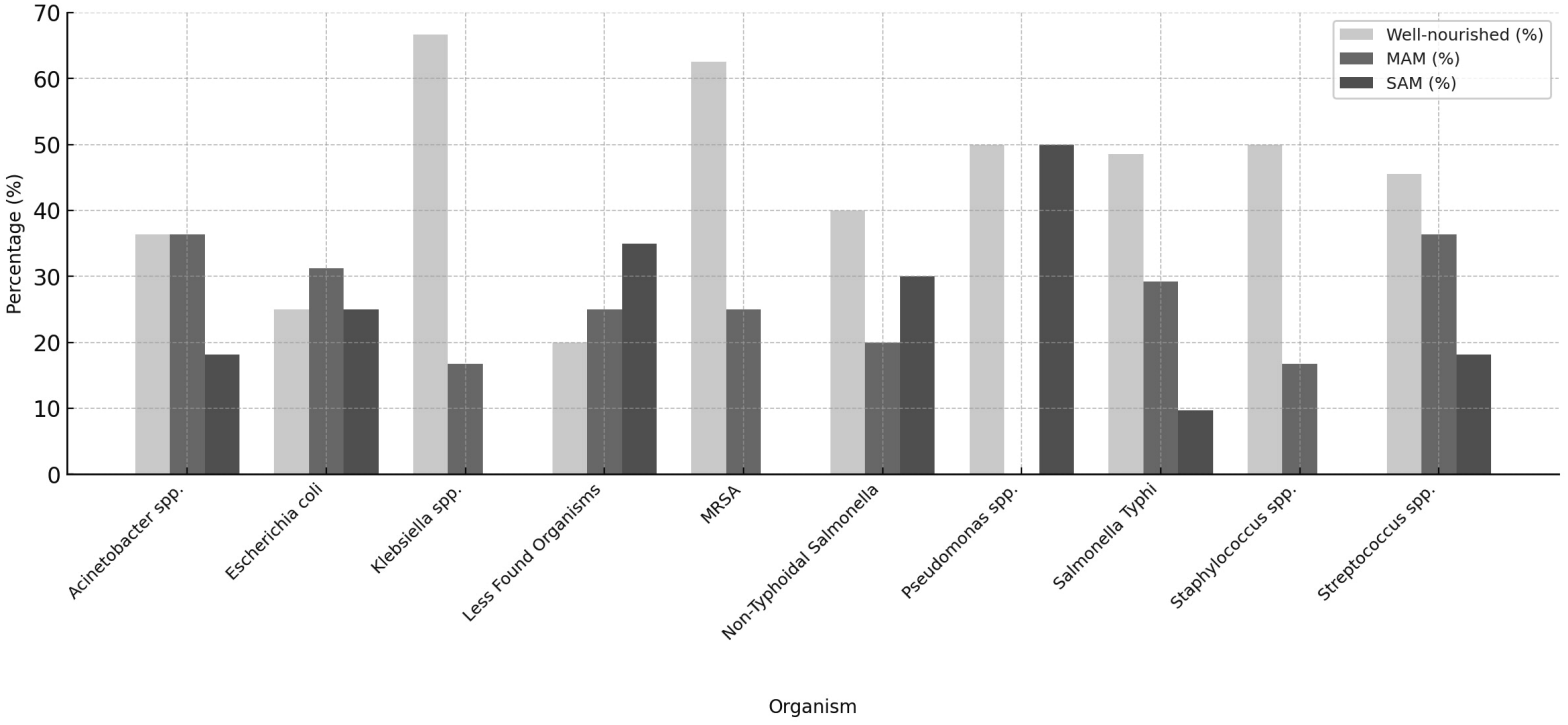
Nutritional status distribution by bacterial pathogen.

The distribution of nutritional status varied across bacterial pathogens (Fig 1). Among children with *Salmonella* Typhi bacteremia, 48.6% were well-nourished, 29.2% had MAM, and 9.7% had SAM. In contrast, higher proportions of malnutrition were observed in non-Salmonella infections: for example, among *Acinetobacter* sp. infections, 36.4% were well-nourished, 36.4% had MAM, and 18.2% had SAM, while 50% of children with *Pseudomonas* sp. infections had SAM. *Klebsiella* sp. infections showed a majority of well-nourished cases (66.7%), whereas *E. coli* infections affected both well-nourished (25.0%) and malnourished (56.2%) children. Overall, *Salmonella* Typhi bacteremia occurred more frequently in well-nourished children, while malnutrition, particularly SAM, was common among other bacterial infections.

Bivariate analysis revealed that nutritional status was a significant factor in determining the pattern of bacteremia among cases and controls. Multivariate logistic regression confirmed that malnourished children (both MAM and SAM) had significantly lower odds of developing *Salmonella* Typhi bacteremia compared to well-nourished children. The adjusted odds ratio (aOR) for SAM was 0.157 (95% CI: 0.045–0.548, p = 0.004), while for MAM, the aOR was 0.238 (95% CI: 0.089–0.640, p = 0.004) (Table 3).

**Table 3:** Association between nutritional status and *Salmonella* Typhi bacteremia.

Nutritional Status	Cases (n = 74)	Controls (n = 88)	Adjusted OR (95% CI)	p-value
Well-nourished	41 (66.13%)	21 (33.87%)	Reference	–
Moderate Acute Malnutrition (MAM)	15 (32.61%)	31 (67.39%)	0.238 (0.089-0.640)	0.004
Severe Acute Malnutrition (SAM)	7 (21.88%)	25 (78.12%)	0.157 (0.045-0.548)	0.004

The mortality rate among children with non-*Salmonella* infections was descriptively higher (~60%) compared to those with *Salmonella* Typhi bacteremia (0%). However, this difference did not reach statistical significance (Chi-square test, p = 1.0), likely reflecting limited sample size and few death events among *Salmonella* cases. Analysis of nutritional status showed that 50.5% of deceased children were well-nourished, whereas severe acute malnutrition (SAM) was more prevalent among survivors (53.8%) (Table 4).

**Table 4:** Nutritional status distribution among deceased vs recovered.

Nutrition Status	Recovered (%)	Deceased (%)
Well-nourished	29.2%	50.5%
MAM	16.9%	24.7%
SAM	53.8%	24.7%

## Discussion

This study explores into the relationship between nutritional status and the patterns of bacteremia in under-five children hospitalized with diarrheal diseases. Our findings demonstrate that while *Salmonella* Typhi bacteremia was significantly more common in well-nourished children, while malnourished children were at greater risk for severe bloodstream infections caused by other pathogens, including *Escherichia coli*, *Klebsiella* spp., *Pseudomonas* spp., and *Acinetobacter* spp.

Our findings align with previous research indicating that malnutrition is a major risk factor for bacterial infections. Studies have shown that malnourished children are more susceptible to invasive bacterial diseases due to impaired immune responses and increased mucosal permeability [18–21]. However, the observed inverse association between *Salmonella* Typhi bacteremia and malnutrition has never been well-documented and warrants further investigation.

The observation that *Salmonella* Typhi bacteremia occurred predominantly in well-nourished children contrasts with previous literature suggesting increased risk of typhoid bacteremia in malnourished children [19,21]. This discrepancy may reflect differences in study populations, pathogen exposure, or health-seeking behavior, and warrants further investigation. It suggests that well-nourished children may not necessarily be protected from invasive *Salmonella* Typhi infection, possibly due to differential exposure patterns in this population.

Although mortality appeared higher in non-*Salmonella* infections, the association was not statistically significant in this cohort, possibly due to small numbers of *Klebsiella* and *E. coli* infections and rare deaths in *Salmonella* Typhi cases. The observation that a larger proportion of deceased children were well-nourished, while SAM was more common among survivors, suggests that mortality risk may be driven more by pathogen virulence than by nutritional status alone [22–24]. These findings highlight that well-nourished children may not be inherently protected from severe outcomes, particularly with opportunistic pathogens, emphasizing the importance of pathogen-specific disease severity over nutritional background in determining outcomes.

This study faces limitations including a retrospective design, a hospital-based population, and a lack of longitudinal data. Future research should address these limitations selecting controls and explore long-term outcomes to better understand bacteremia in malnourished children.

This study provides new evidence that malnutrition influences bacteremia patterns in children with diarrheal diseases. While well-nourished children were more likely to develop *Salmonella* Typhi bacteremia, malnourished children exhibited higher susceptibility to bacterial infections, including Non-Typhoidal *Salmonella, Escherichia coli, Streptococcus* spp., *Staphylococcus* spp., MRSA, *Klebsiella* spp., *Pseudomonas* spp., and *Acinetobacter* spp*.* leading to increased mortality. These distribution of bacteremia patterns across patients’ nutritional status can provide insights and improve clinical management. These findings highlight the need for integrated strategies combining nutritional support, infection control, and antimicrobial stewardship to reduce the burden of bloodstream infections in vulnerable pediatric populations. Future research should focus on elucidating the biological mechanisms underlying these associations to inform targeted interventions and policy recommendations.

## References

[1] Frenkel L. Infectious diseases as a cause of global childhood mortality and morbidity: Progress in recognition, prevention, and treatment. Adv Pediatr Res. 2018;5:1–11.

[2] Kotloff KL. The Burden and Etiology of Diarrheal Illness in Developing Countries. Pediatr Clin North Am. 2017;64(4):799–814. doi: 10.1016/j.pcl.2017.03.006 28734511

[3] Butler T, Bell WR, Levin J, Linh NN, Arnold K. Typhoid fever. Studies of blood coagulation, bacteremia, and endotoxemia. Arch Intern Med. 1978;138(3):407–10. doi: 10.1001/archinte.138.3.407 629635

[4] Marchello CS, Birkhold M, Crump JA. Complications and mortality of typhoid fever: A global systematic review and meta-analysis. J Infect. 2020;81(6):902–10. doi: 10.1016/j.jinf.2020.10.030 33144193 PMC7754788

[5] UNICEF. Child Malnutrition. UNICEF; 2023. Available from: https://data.unicef.org/topic/nutrition/malnutrition/

[6] Chandra RK. Influence of Nutritional Status on Susceptibility to Infection. Adv Nutrit Res. 1979:57–77. doi: 10.1007/978-1-4613-9931-5_3

[7] Bari A, Nazar M, Iftikhar A, Mehreen S. Comparison of Weight-for-Height Z-score and Mid-Upper Arm Circumference to Diagnose Moderate and Severe Acute Malnutrition in children aged 6-59 months. Pak J Med Sci. 2019;35(2):337–41. doi: 10.12669/pjms.35.2.45 31086511 PMC6500831

[8] Jones KD, Thitiri J, Ngari M, Berkley JA. Childhood malnutrition: toward an understanding of infections, inflammation, and antimicrobials. Food Nutr Bull. 2014;35(2 Suppl):S64-70. doi: 10.1177/15648265140352S110 25069296 PMC4257992

[9] O’Sullivan NP, Lelijveld N, Rutishauser-Perera A, Kerac M, James P. Follow-up between 6 and 24 months after discharge from treatment for severe acute malnutrition in children aged 6-59 months: A systematic review. PLoS One. 2018;13(8):e0202053. doi: 10.1371/journal.pone.0202053 30161151 PMC6116928

[10] Jones KDJ, Berkley JA. Severe acute malnutrition and infection. Paediatr Int Child Health. 2014;34(Suppl 1):S1–29. doi: 10.1179/2046904714Z.000000000218 25475887 PMC4266374

[11] Bahwere P, Levy J, Hennart P, Donnen P, Lomoyo W, Dramaix-Wilmet M, et al. Community-acquired bacteremia among hospitalized children in rural central Africa. Int J Infect Dis. 2001;5(4):180–8. doi: 10.1016/s1201-9712(01)90067-0 11953214

[12] Wolf BH, Ikeogu MO, Vos ET. Effect of nutritional and HIV status on bacteraemia in Zimbabwean children who died at home. Eur J Pediatr. 1995;154(4):299–303. doi: 10.1007/BF01957366 7607281

[13] Ahs JW, Tao W, Löfgren J, Forsberg BC. Diarrheal Diseases in Low- and Middle-Income Countries: Incidence, Prevention and Management. Open Infect Dis J. 2010;4(1):113–24. doi: 10.2174/1874279301004010113

[14] Thaxton GE. Moderate acute malnutrition: inflammatory response, microbiota, and potential treatments. 2019.

[15] Rao S, Schieber AMP, O’Connor CP, Leblanc M, Michel D, Ayres JS. Pathogen-Mediated Inhibition of Anorexia Promotes Host Survival and Transmission. Cell. 2017;168(3):503-516.e12. doi: 10.1016/j.cell.2017.01.006 28129542 PMC5324724

[16] Fontaine F, Turjeman S, Callens K, Koren O. The intersection of undernutrition, microbiome, and child development in the first years of life. Nat Commun. 2023;14(1):3554. doi: 10.1038/s41467-023-39285-9 37322020 PMC10272168

[17] Kau AL, Ahern PP, Griffin NW, Goodman AL, Gordon JI. Human nutrition, the gut microbiome and the immune system. Nature. 2011;474(7351):327–36. doi: 10.1038/nature10213 21677749 PMC3298082

[18] Bhutta ZA. Typhoid fever: current concepts. Infect Dis Clin Pract. 2006;14:266–72.

[19] Chisti MJ, Hossain MI, Malek MA, Faruque ASG, Ahmed T, Salam MA. Characteristics of severely malnourished under-five children hospitalized with diarrhoea, and their policy implications. Acta Paediatr. 2007;96(5):693–6. doi: 10.1111/j.1651-2227.2007.00192.x 17462060

[20] Griffiths JK. Malnutrition and undernutrition. In: Water and sanitation‐related diseases and the environment: Challenges, interventions, and preventive measures. 2011. p. 71–80.

[21] Ulijaszek SJ. Relationships between undernutrition, infection, and growth and development. Hum Evol. 1996;11(3–4):233–48. doi: 10.1007/bf02436627

[22] Nalwanga D, Musiime V, Kizito S, Kiggundu JB, Batte A, Musoke P, et al. Mortality among children under five years admitted for routine care of severe acute malnutrition: a prospective cohort study from Kampala, Uganda. BMC Pediatr. 2020;20(1):182. doi: 10.1186/s12887-020-02094-w 32331517 PMC7181483

[23] Munthali T, Jacobs C, Sitali L, Dambe R, Michelo C. Mortality and morbidity patterns in under-five children with severe acute malnutrition (SAM) in Zambia: a five-year retrospective review of hospital-based records (2009–2013). Arch Public Health. 2015;73:1–9. 25937927 10.1186/s13690-015-0072-1 PMC4416273

[24] Attia S, Versloot CJ, Voskuijl W, van Vliet SJ, Di Giovanni V, Zhang L, et al. Mortality in children with complicated severe acute malnutrition is related to intestinal and systemic inflammation: an observational cohort study. Am J Clin Nutr. 2016;104(5):1441–9. doi: 10.3945/ajcn.116.130518 27655441 PMC5081715

